# Shape fidelity and structure of 3D printed high consistency nanocellulose

**DOI:** 10.1038/s41598-019-40469-x

**Published:** 2019-03-07

**Authors:** Ville Klar, Jaakko Pere, Tuomas Turpeinen, Pyry Kärki, Hannes Orelma, Petri Kuosmanen

**Affiliations:** 1 Aalto University School of Engineering, Department of Mechanical Engineering, Aalto, 00076 Finland; 2 VTT Technical Research Centre of Finland Ltd, Espoo, 02044 Finland

**Keywords:** Mechanical engineering, Mechanical engineering, Mechanical engineering, Mechanical engineering, Bioinspired materials

## Abstract

The aim of the present study was to investigate the additive manufacturing process for high consistency nanocellulose. Unlike thermoformable plastics, wood derived nanocelluloses are typically processed as aqueous dispersions because they are not melt-processable on their own. The ability to use nanocellulose directly in additive manufacturing broadens the possibilities regarding usable raw materials and achievable properties thereof. Modern additive manufacturing systems are capable of depositing nanocellulose with micrometer precision, which enables the printing of accurate three-dimensional wet structures. Typically, these wet structures are produced from dilute aqueous fibrillar dispersions. As a consequence of the high water content, the structures deform and shrink during drying unless the constructs are freeze-dried. While freeze-drying preserves the geometry, it results in high porosity which manifests as poor mechanical and barrier properties. Herein, we study an additive manufacturing process for high consistency enzymatically fibrillated cellulose nanofibers in terms of printability, shape retention, structure, and mechanical properties. Particular emphasis is placed on quantitative shape analysis based on 3D scanning, point cloud analysis, and x-ray microtomography. Despite substantial volumetric as well as anisotropic deformation, we demonstrate repeatability of the printed construct and its properties.

## Introduction

In the past decades, additive manufacturing (AM) technologies have become commonplace both in industry and home use. Material extrusion based additive manufacturing, often termed three-dimensional (3D) printing, is an appealing manufacturing technique as it enables raw material efficient semi-automated, toolless and patternless manufacturing of complex geometries directly from models produced with computer-aided design (CAD)^1–3^. Thermoplastic materials, such as petrochemical polymers and metals, are widely used in 3D printing. Despite the recyclability of some common thermoplastic AM feedstocks, many of them are associated with severe adverse environmental impacts such as the accumulation of plastic waste in the marine environment^4,5^. To reduce the dependency on petrochemical materials, many research groups have shifted their focus to the development of various biomaterials in AM.

As an abundant and performant biopolymer, cellulose has been intensively researched as a raw material for 3D printing. The diverse semi-crystalline fiber morphologies of cellulose found in nature exhibit remarkable properties in terms of tensile strength, stiffness and thermal stability^6–8^. However, it requires considerable engineering effort to truly capitalize on these properties in man-made structures.

Various printing approaches and feedstock formulations for cellulosic raw materials have been extensively reviewed^9–12^. In broad terms, the cellulose based raw materials suitable for AM can be divided into three categories: regenerated, derivatives, and native cellulose based materials. Feedstock formulations for AM have been proposed from all three categories. Regeneration based AM for cellulose is not ideal as sufficient desolvation needs to be carefully balanced with adequate layer adhesion to achieve stable 3D structures. Cellulose derivatives have potential as an AM feedstock but the microfibrillar structure along with other advantageous properties such as thermal stability are typically lost during derivatisation. The main benefits of native cellulose materials are their great modification potential, excellent biodegradability, as well as high thermal and electrical resistance. However, the lack of thermoformability constrains the use of native cellulose based materials on their own in common AM techniques such as fused filament fabrication (FFF).

An alternative route, that does not require thermoformability, is to 3D print native cellulose based materials with techniques based on the extrusion of hydrogels or solutions. One of the most commonly utilized techniques is direct ink writing (DIW), also known as direct paste writing, paste extrusion, or robocasting. DIW is a programmable AM technique based on the layerwise extrusion of moldable liquids or pastes, often termed inks. In addition to native cellulose fiber suspensions, DIW has been utilized to 3D print cellulose solutions^13–15^ as well as cellulose derivatives^16–19^.

Arguably, most DIW research with native cellulose materials has been performed with inks consisting of nanostructured cellulose commonly known as nanocellulose. Nanocellulose forms thick shear-thinning gels already at low consistencies which facilitates the printing of high water content freestanding constructs^20,21^. One of the most notable applications of AM nanocellulose is biofabrication. Nanocellulose is suitable for bioinks in applications including tissue engineering^22,23^ and wound dressings^24,25^.

The nanocellulose used in this research is produced with a novel enzyme assisted high consistency fibrillation method (HefCel)^26,27^. The HefCel process enables production of enzymatically fibrillated cellulose nanofibers (EFCNF) from softwood pulp. EFCNF differs from other types nanocellulose with its strong adhesion properties and ability to be processed at high consistencies. Furthermore, the utilized enzymatic pretreatments are similar to cellulose degradation mechanisms found in nature and they are considered to have a lower environmental impact compared to equivalent chemical and mechanical fibrillation methods^28^. All of these attributes are advantageous in AM of native cellulose constructs and encourage exploring the AM of high consistency EFCNF.

While a low consistency is beneficial in biofabrication, it is detrimental if the printed structure needs to be dried. Native cellulose fiber constructs undergo substantial deformation during drying as the water evaporates or flows out from the structure. Many studies resort to freeze-drying to prevent collapse and retain the intended shape in the 3D prints^29–31^. However, freeze-drying compromises mechanical properties. Håkansson *et al*.^31^ compared different drying methods for cellulose nanofiber (CNF) constructs. They showed, that while freeze-drying can be used to preserve geometry it results in a 97% reduction in ultimate tensile strength and 99% reduction in stiffness compared to air dried samples. Conversely, the drying deformation of air dried samples was substantially larger.

**Figure 1:**
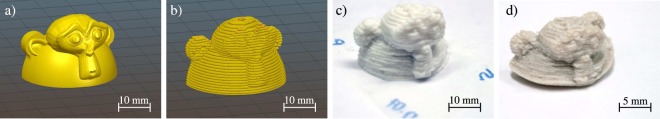
Drying distortion of a print based on the test model “Suzanne” from Blender^47^. (**a**) Original mesh, (**b**) Rendering of printing path with 0.84 mm diameter syringe, (**c**) Wet printed structure, (**b**) Dry printed structure.

As demonstrated by Håkansson *et al*.^31^ air drying of AM constructs produced from low consistency nanocellulose results in poor shape retention. However, by increasing the consistency and using appropriate air drying conditions, the drying deformation can be reduced. The constructs shrink and collapse substantially but the original design intent is present in the final dry construct. An example of the air drying deformation with high consistency EFCNF is shown in Fig. 1.

Instead of freeze-drying, we used high consistency printing pastes (from 15.5 wt% to 25 wt%) and air drying in controlled temperature (T) and relative humidity (RH) in an effort to promote shape retention. In this study we evaluate shape retention as the retention of the original shape primitive. We allow both volumetric and collapse as they cannot be fully avoided with the chosen drying method. We quantify the extent and repeatability of these deformations and report on the influence of air drying conditions and consistency on shape retention, porosity and tensile properties. We demonstrate the repeatability and controllability of the structure and properties of air dried AM EFCNF constructs. Such predictability paves the way for designers to know how much the AM EFCNF structures deform during drying and take this into account when designing parts. Ultimately, this work introduces a new viable route to produce and analyze pure cellulosic AM constructs with unique properties.

## Results

### Printability of high solids content EFCNF

Despite active research on the printability of various bioinks, the term lacks a formal definition and standardized evaluation methods^11^. In the context of this paper, we consider printability to be the combination of two factors: extrudability and the ability to form freestanding wet constructs with a layerwise approach. EFCNF paste fulfils the requirements of a DIW-suitable material by behaving like a nonviscous liquid during extrusion and a shape stable gel once it has been deposited in the desired place.

Leppiniemi *et al*.^24^ studied a nanocellulose-alginate hydrogels and performed computational fluid dynamic simulation on the extrusion and printing process. They found that printability is sensitive to the rheological properties of the hydrogel. Rheological properties of nanocellulose suspensions depend significantly on factors such as fiber dimensions, surface character and consistency^32^. In the context of this research we investigated the rheological aspects of printability of high consistency EFCNF through trial and error. We found that the consistency range of 15.5 to 25 wt% was extrudable with the chosen printing technique and parameters.

The two lower consistencies (15.5 and 20 wt%) exhibited a lower clogging propensity as well as a more stable extrusion pressure. While the extrusion pressure is not a direct measure of volumetric flow rate with shear thinning fluids, its stability indicates a steady volumetric flow. The two lower consistencies were printable with a luer tip syringe with a 0.84 mm diameter which enables a higher printing resolution.

The 25 wt% suspensions exhibited a high clogging propensity even with a 1.2 mm syringe tip. Enzymatic CNF suspensions are known to exhibit fibrillar flocs with sizes ranging between 50 µm and 1000 µm^33,34^. The average extrusion pressures at a 0.56 ml/min flow rate with the 1.2 mm syringe tip for the 15.5 wt%, 20 wt% and 25 wt% suspensions were approximately 1 bar, 3 bar and 10 bar respectively. The extrusion pressure fluctuated more with 25 wt% pastes indicating an unsteady volumetric flow rate. Such fluctuation has an adverse effect on the printing as an uneven amount of material is deposited.

To enable consistent extrusion of high consistency EFCNF, we used a purpose-built extruder. We found that extrusion systems based on pressurized air or feed screws perform poorly in the extrusion of high consistency EFCNF. Firstly, the compressibility of air results in a spring-like actuation of the syringe piston. This spring-like behavior results in inaccurate dosing. Secondly, valve operated flow control seizes to be reliable at higher consistencies as the larger fiber agglomerates readily clog the valve inlet. Feed screw and progressive cavity based systems suffered from similar clogging issues at higher consistencies. The custom extruder system used in this research is based on direct mechanical actuation of the piston syringe, without high-pressure air or micro-valves. The extruder is similar to the piston based DIW extruder described by Xu *et al*.^12^, but it features additional sensors for piston position and force which enables precise dosing. This direct actuation combined with the force sensor and linear position feedback enables precise dosing of high consistency EFCNF. The extruder design is described and discussed in further detail in the Supplementary Section.

Once extruded, the EFCNF pastes exhibit a high level of wet shape retention. Even with the comparatively low infill density of 50% the shapes did not collapse under their own weight during printing. The 50% infill density with a rectilinear infill pattern, two perimeters and two solid top and bottom layers results in a 82% ratio of extruded volume to model volume with the cubic test geometry. The ability to utilize different infill densities and patterns means that the porosity and its distribution can be influenced with printing parameters.

At the highest consistency (25 wt%) we observed a decrease in the adhesion propensity of the paste. The paste seized to adhere properly to layers below it and to the printed paths next to it. The reduction in adhesion propensity especially challenging during the first layers of printing. Similarly to other layerwise extrusion processes, the first layer needs to properly adhere to the printbed in order for subsequent layers to be accurately deposited on top of it. The 25 wt% suspensions exhibited a lower adhesion propensity compared to the two lower consistency suspensions. This meant that the margin of error in setting the initial layer height for the highest consistency was lower compared to the two lower consistencies.

### Drying rate and deformation

**Figure 2:**
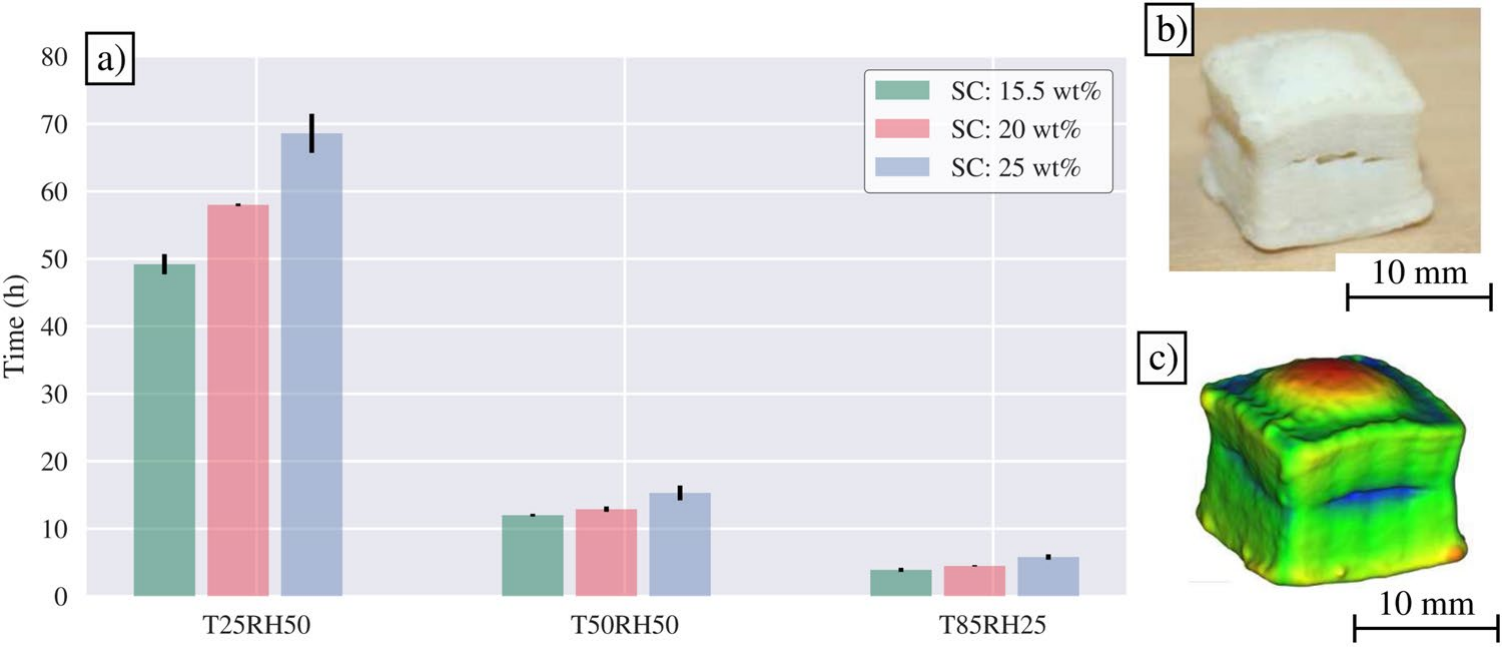
(**a**) Bar graph showing the drying times of the tested samples, (**b**) Photograph of cracked sample (20 wt% EFCNF dried at 85 °C and 25% relative humidity), (**c**) Visualization of drying deformation of sample shown in Fig. 2b, where red, green and blue colors denote positive, low and negative error from a fitted box-like shape respectively.

Drying times are shown in Fig. 2a. More detailed drying rate data as well as the fitting is shown in the Supplementary Section (Figs S2, S3 and S4). The drying curves exhibit a similar linear and falling rate phases as reported in the drying phases of papermaking^35^.

As expected, increasing temperature and reducing humidity decreases drying times with an average drying time of 59 h for T: 25 °C, RH: 50%, 13 h for T:50 °C, RH: 50% and 5 h for T: 85 °C, RH: 25% A higher initial consistency increases the drying time but has a less pronounced influence compared to drying conditions. For example at T: 50 °C and RH: 50%, the drying time for 15.5 wt%, 20 wt% and 25 wt% cubes was on average 12 h, 13 h and 15 h. The drying times measured in this research are roughly in the same scale as reported for die-cast microfibrillated cellulose (MFC) films^36^.

Increasing the drying rate had adverse effects on the geometrical fidelity of the 3D prints. Most notably the prints started to crack and bulge. Cracking and bulging was observed with all consistencies with 85 °C and 25% relative humidity drying. Some cracking was also observed with constructs printed with the highest consistency (25 wt%) dried at 50 °C and 50% relative humidity. The observations suggest that cracking propensity is influenced both by consistency and drying conditions. The cracking and bulging of sample SC20T85RH25 is illustrated in Fig. 2b,c.

A similar drying deformation is a well-known in the context of cellulose based paper and film, where unevenness in moisture typically results in stretched edges, wrinkles and web breaks. Once formed, the cracks are likely to increase in size as water vapor finds its way through the structure along the path with the least resistance^37^. Uneven drying is disadvantageous both in terms of structural integrity and geometrical fidelity. In most samples the crack started to form in the middle of the print and the bulging at the top as shown in Fig. 2b,c.

In previous research the deformation and shape analysis regarding AM cellulose constructs has been assessed with manual measurement tools^21^ or qualitative visual inspection^31^. Li *et al*.^29^ utilized an image analysis technique performed on 1 cm^3^ cubes for print quality evaluation. While these techniques provide estimates on shape and dimensions, they do not adequately capture the 3D nature of AM constructs. We extend this work by introducing a shape retention evaluation method based on 3D scanning. This method enables a more detailed classification of the various deformation types associated with air drying of AM cellulose constructs. The scanning process, point cloud analysis as well as an error analysis of the procedure are described in further detail in the Supplementary Information of this paper.

**Figure 3:**
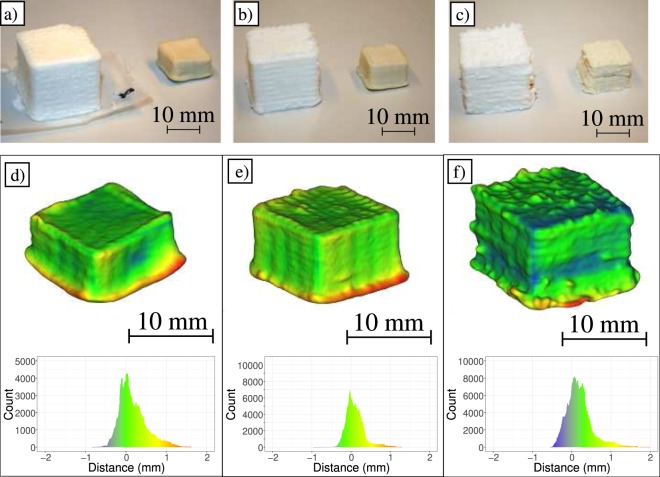
Printed constructs before and after drying. (**a**) SC15.5T25RH50, (**b**) SC20T25RH50, (**c**) SC25T25RH50. Deviation from fitted shape primitive illustrated in colors, where red, green and blue colors denote positive, low and negative deviation respectively. The distance distribution from the fitted shape primitive is shown below the corresponding sample. (**d**) SC15.5T25RH50, (**e**) SC20T25RH50, (**f**) SC25T25RH50.

Figure 3a–c shows the dried constructs (T:25 °C, RH:50%) next to their wet counterparts. While the structures are significantly deformed there are no severely adverse defects such as large cracks. We measured the principal dimensions of the constructs (X, Y and Z) by fitting planes on each surface and calculating the mean distances between two opposing planes. The volumes are computed from the models using the divergence theorem^38^. Table 1 shows the volume (V) and the dimensions between fitted planes of the surfaces each Cartesian direction (X, Y and Z) with Z being the stratification direction in the printing process.

**Table 1:** Dimensions of printed cubes, reported error values are the standard error calculated from measurements performed on three parallel measurements.

Sample name	V (ml)	X (mm)	Y (mm)	Z (mm)	RMS (mm)	d (mm)	SD_d_ (mm)
SC15.5T25RH50	1.3 ± 0.07	12.9 ± 0.4	12.8 ± 0.5	6.9 ± 0.4	0.4 ± 0.04	0.2 ± 0.02	0.34 ± 0.04
SC15.5T50RH50	1.3 ± 0.08	12.8 ± 0.4	12.5 ± 0.4	7.37 ± 0.2	0.39 ± 0.03	0.17 ± 0.03	0.34 ± 0.02
SC15.5T85RH25	1.5 ± 0.1	12.4 ± 0.5	12.9 ± 0.5	8.63 ± 0.4	0.52 ± 0.05	0.26 ± 0.04	0.45 ± 0.04
SC20T25RH50	1.7 ± 0.006	13 ± 0.2	13.1 ± 0.2	9.29 ± 0.2	0.26 ± 0.003	0.11 ± 0.003	0.24 ± 0.003
SC20T50RH50	1.7 ± 0.03	12.9 ± 0.3	12.7 ± 0.3	9.71 ± 0.3	0.32 ± 0.02	0.16 ± 0.03	0.3 ± 0.02
SC20T85RH25	2.0 ± 0.04	12.8 ± 0.5	12.9 ± 0.4	10.9 ± 0.4	0.43 ± 0.004	0.25 ± 0.008	0.35 ± 7e-04
SC25T25RH50	2.1 ± 0.2	13.9 ± 0.3	13.8 ± 0.3	10.4 ± 0.2	0.32 ± 0.06	0.15 ± 0.03	0.29 ± 0.05
SC25T50RH50	2.3 ± 0.1	13.8 ± 0.5	13.7 ± 0.4	10.9 ± 0.3	0.46 ± 0.07	0.26 ± 0.04	0.38 ± 0.06
SC25T85RH25	2.7 ± 0.04	14.7 ± 0.4	14.6 ± 0.4	11.9 ± 0.4	0.48 ± 0.1	0.22 ± 0.02	0.42 ± 0.1

To describe how well the X, Y and Z dimensions correspond with the scanned point cloud, we performed a fitting algorithm with box-like shape primitive with the same X, Y and Z dimensions. The root-mean-square (RMS) of the fit as well as the mean distance (d) between the fitted shape primitive and the point cloud is also shown in Table 1. The mean standard deviation (SD_d_) of the distance is calculated from the three parallel samples.

The distance between the fitted primitive and point cloud is illustrated in Fig. 3d–f where geometrical error between the fitted box and the point cloud is visualized with colors. Negative errors (meaning inside the fitted primitive) are shown in blue and positive errors (meaning outside the fitted primitive) are shown in red. The green color denotes a low error between the scanned point cloud and the fitted primitive. The distance distribution from the fitted shape primitive is also shown below the corresponding sample. A similar visualization is provided for all the scanned samples in the Supplementary Section Figs S7, S8 and S9.

Air drying results in two distinct forms of deformation: volumetric deformation and collapse. While these deformations were observed in all samples, the standard error values shown in Table 1 suggests that they are repeatable. As Fig. 3 and Table 1 illustrate the most apparent form of deformation is volumetric. The volumes are reduced proportionately to consistency with samples printed with 15.5 wt%, 20 wt% and 25 wt% EFCNF reducing on average to approximately 16%, 21% and 27% of the original model volume respectively. Comparing the two slower drying conditions (T:25 °C; RH 50% and T:50 °C; RH:50%) the volumetric shrinkage is reduced with faster drying. The volumetric shrinkage in samples printed with 15.5 wt%, 20 wt% and 25 wt% EFCNF was reduced on average by 3%, 3.5% and 7.4% by increasing the temperature from 25 °C to 50 °C with the same relative humidity. While the trend continues to the fastest drying (T: 85 °C, RH: 50%), we do not consider these samples passable as they all had large cracks and other geometrical distortions.

The second most apparent deformation is the anisotropic shrinkage in the stratification or Z - direction which we denote as collapse. This phenomenon has been reported by several research groups^24,29,31^ and it is commonly avoided through freeze-drying. Table 1 shows that similarly to volumetric change the collapse decreases both by increasing consistency and increasing the drying rate. Samples that were dried at slower drying rates exhibited larger X and Y dimensions in relation to the Z-dimensions. In other words, a moderate increase of the drying rate results in a moderate reduction of collapse. For example, the Z dimensions of samples printed with 15.5 wt%, 20 wt% and 25 wt% EFCNF were on average 7.2%, 4.5% and 4.8% higher in samples dried at 50 °C compared to samples dried at 25 °C with the same relative humidity. It should be noted that the bulging of the top surface associated with the fastest drying rate (T:85 °C, RH:25%) results in high values for the Z dimensions in those samples. Consequently, these values should not be interpreted solely as a reduction in collapse.

These two deformations, volumetric deformation and collapse, are expected in air drying. Thusly the expected output with a cube input is a box-like shape with a smaller height (Z) compared to width and length (X and Y). RMS, d and SD_d_ values reported in Table 1 quantify the remaining shape error after volumetric deformation and collapse have been taken into account. There are two types of shape error that contribute to high RMS, d and SD_d_ values which we denote as warping and roughness.

Warping is illustrated in Fig. 3d, where the contorted surfaces of the box-like shape printed from 15.5 wt% are shown. Samples printed with the 15.5 wt% EFCNF exhibited a higher degree of such contortion compared to samples printed with 20 wt% or 25 wt% EFCNF. Another distinct type of warping is sagging of the shape. It refers to the widening of the bottommost layers of the printed construct. It is shown in Fig. 3d–f as the red areas around the bottom of the structure. Such sagging has also been observed with AM cellulose nanocrystal (CNC) constructs^29^. Sagging reduces with increased consistency but it was present in all scanned samples.

The other shape error, roughness, refers to small (0.1 to 0.2 mm diameter) protrusions on the external surface of the structure (Fig. 3f). Roughness was most apparent with the highest consistency (25 wt%) and appeared to decrease with consistency. The results indicate that roughness is mainly related to consistency rather than drying parameters.

Based on the standard error of of the X, Y and Z dimensions, a low RMS as well as low distance and standard deviation thereof, the lowest shape error was achieved with samples dried at 25 °C and RH: 50%. The highest degree of warping was measured from samples dried at T: 85 °C and RH: 25% as a result of the bulging and cracking. The smallest standard error in dimensions and RMS value was observed with samples printed with 20 wt% EFCNF and dried at 25 °C and RH: 50%.

### XµCT analysis

The internal 3D structure of the printed cubes was determined using XµCT, which is an imaging method for obtaining a digitalized 3D density map of virtually any kind of material^39^. It is an optimal tool for studying the structure and the internal material composition of fragile samples or samples that can not be cut.

A total of three samples were analyzed with XµCT, one sample from each of the tested consistencies (15.5 wt%, 20 wt% and 25 wt%). All the samples analyzed with XµCT were dried at 50 °C and RH: 50%. The porosity for samples printed from 15.5 wt%, 20 wt% and 25 wt% EFCNF was 12.4%, 14.4% and 16.7% respectively. The results indicate that as the initial consistency is increased the porosity of air dried construct increases. An opposite correlation has been reported with freeze-dried 3D printed CNC constructs^30^. In freeze-drying based approaches large pores are inevitable^31^.

The XµCT data suggests that the correlation between loading and porosity stems from the 50% infill density. Higher consistency EFCNF is more viscous which means that the wet matrix of the printed construct is more rigid and consequently less prone to collapse. As we show, the higher consistencies exhibit a lower volumetric deformation compared to lower consistencies. Thusly, the construct retains a higher amount of the initial internal void space.

**Figure 4:**
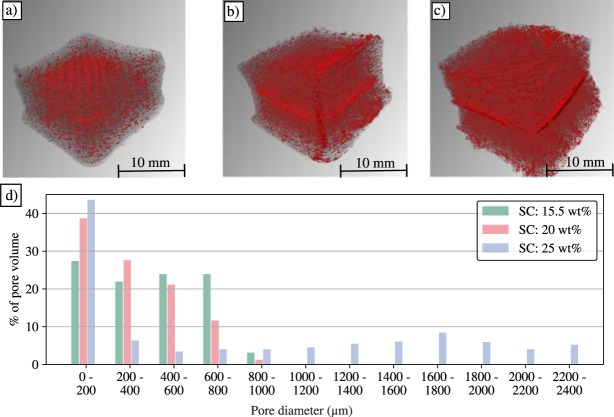
3D visualizations of the XµCT images, where void space is shown as red. (**a**) 15.5 wt%, (**b**) 20 wt%, (**c**) 25 wt%. (**d**) Pore size distribution.

The 3D-visualizations (Fig. 4a–c) show the void space within the printed constructs. The red color denotes void space and the semi-transparent gray color denotes solid space. In samples printed from 15.5 wt% EFCNF the pores are aligned according to the infill pattern. The amount of pores and larger void areas is highest in the innermost parts of the geometry and their size and amount decreases toward the exterior. While large porous areas were observed in samples printed from 20 wt% and 25 wt% as well, the pores appear more uniformly distributed within the structures in those samples.

The 3D visualizations show larger void sections the middle of the sample printed from 25 wt% (Fig. 4c). While less dense, similar sections are visible in the sample printed from 20 wt% EFCNF(Fig. 4b). Similarly to the correlation between loading and porosity, the formation of these porous areas was attributed to infill parameters. This suggests that the internal geometry can be controlled with the infill parameters despite air drying related deformations.

The pore size distribution is shown in Fig. 4d. The total pore volume is more evenly distributed with pores up to 1 mm in size in the samples printed from 15.5 wt% compared to the 20 wt% and 25 wt% samples. In the sample printed with 25 wt% EFCNF approximately half of the total pore volume is in pores with diameters less than 200 µm. It also exhibited the largest pores with diameters exceeding 2 mm. Samples printed from 15.5 wt% and 20 wt% EFCNF did not exhibit pores larger than 1 mm in diameter.

### Tensile properties

The influence of consistency on tensile properties of the DIW constructs was studied via stress-strain measurements. The mean stress, stiffness, load and elongation at break as well density of tensile testing specimens are shown in Table 2. Stress-elongation curves of the 3D printed structures are shown in the Supplementary Section (Fig. S12). In this study, we used small non-standard geometries for tensile testing (shown in the Supplementary Section Fig. S11). The non-standard geometries were chosen to reduce curling. This curling tendency is illustrated (Fig. S10) and discussed in further detail in the Supplementary Section.

**Table 2:** Tensile properties at break, reported error values are the standard error calculated from measurements performed on five parallel measurements.

	SC15.5T50RH50	SC20T50RH50	SC25T50RH50
Stress (MPa)	31.1 ± 1.7	25.2 ± 3.1	17.6 ± 3.4
Stiffness (MPa)	329.2 ± 78	503.4 ± 44.7	440.1 ± 68.1
Load at break (N)	572 ± 32.2	646.6 ± 60.4	651 ± 129.6
Elongation (%)	9.2 ± 1.2	9.7 ± 0.5	6.6 ± 0.7
Density (g/cm^3^)	1.31 ± 0.02	1.24 ± 0.05	1.20 ± 0.3

Samples printed with 15.5 wt% EFCNF exhibited the highest tensile strength and samples printed with the 25 wt% EFCNF the lowest. The elongation at break of samples printed with the 15.5 wt% and 20 wt% EFCNF was approximately 30% higher than the samples printed with the 25 wt% EFCNF. The results indicate that a lower consistency is more favourable for strength development compared to a higher consistency.

Most additively manufactured thermoplastic polymers have tensile strengths in the range of 30–40 MPa elongation at break in the range of 5–10%, stiffness in the range of 1 to 2 GPa and densities in the range of 1 to 1.4 g/cm^3^ ^40^. Apart from stiffness, the EFCNF constructs produced in this study are in the same range. Cellulose-based AM constructs typically exhibit inferior properties, for example in terms of strength, compared to counterparts produced via e.g., casting based processes^11^. Die-cast or vacuum filtered MFC films have tensile strengths in the range of 70–300 MPa elongation at break in the range of 2–15% and stiffness values in the range of 3 to 17 GPa^41^. The AM constructs produced in this study exhibit lower values compared to these films. This is likely the result of a lower density compared to vacuum filtered constructs as well as manufacturing process related defects introduced during layerwise extrusion and drying.

While AM of various native cellulose fiber suspensions has been actively researched, the number of reports on tensile properties of air dried constructs is low. The tensile properties of AM nanocellulose constructs has been studied by Håkansson *et al*.^31^. They used a two layer film to approximate the tensile properties achievable with AM nanocellulose constructs. They reported tensile strengths as high as 114 ± 14 MPa and mean stiffness of 4.3 ± 0.3 GPa for air dried samples. More recently native cellulose has been combined with chitosan to produce raw materials suitable for large scale AM^42^. They reported a tensile strength of 11.3 ± 0.6 MPa and a mean stiffness of 244.1 ± 24 MPa for the 3D printed structures.

The mechanical properties align with the porosity of the tested samples with the lowest porosity samples displaying the highest tensile strength. A higher porosity results in lower ultimate stress and stiffness. A similar correlation between porosity and tensile strength has been reported with cellulose nanopaper structures^43^.

## Discussion

3D-structures were successfully printed from EFCNF pastes with different consistencies and drying conditions. The influence of consistency and printing parameters was discussed. Different types of geometrical errors introduced during drying were identified and described. A 3D-scanning based analysis was demonstrated to produce more quantitative results of the 3D shape. XµCT measurements revealed that given a 50% infill pattern, higher consistency results in higher porosity compared structures printed with lower consistencies. Tensile measurement confirmed that air drying results in constructs with similar mechanical properties to AM thermoplastics.

Increasing the consistency and decreasing the drying rate reduces the drying deformation. While the structures undergo a substantial reduction in volume and a collapse in the stratification direction, they retain a box-like shape. The original design intent is present in the shape despite geometrical deformation. The results suggest that a satisfactory degree of shape fidelity and preservation of original design intent can be achieved with air drying and high consistencies. Such findings encourage further research into systematic improvement of process parameters as well as geometry compensation based corrections of the model data. While structural integrity achieved via conventional methods such as die-casting, thermopressing or pressure filtration remains higher, the advantages achievable with AM justify further research.

## Materials and Methods

### Preparation of EFCNF

Never-dried bleached softwood pulp from a Finnish mill (MetsäFibre Oy, Äänekoski, Finland) was used as the raw material for preparation of EFCNF. A high consistency aqueous suspension of fibrillated nanocellulose was prepared as described previously Hiltunen *et al*.^26^. The enzymatic treatment was carried out at a consistency of 25% dry weight for 6 h at 70 °C, pH 5 using a two-shaft sigma mixer (Jaygo Incorporated, NJ, USA) running at 25 rpm. After the treatment, the temperature was raised to 90 °C for 30 min to inactivate the enzyme. The fibrillated material was diluted with deionized water, filtered, and washed thoroughly with deionized water. Finally, the fibrillated material was dewatered to the desired consistencies (15.5, 20 and 25 wt%) by filtration. The material was stored at 4 °C until use. The dry consistency for each suspension was determined by complete drying followed by weighing with a precision scale. The complete drying was performed by keeping the sample in an oven at 105 °C overnight (16 h). The consistency was calculated using Eq. 1,

1 $${C}_{m}=\frac{{m}_{d}}{{m}_{d}+{m}_{w}}\cdot 100 \% $$

where *C*_*m*_ is consistency, *m*_*d*_ is the mass of dry materials and *m*_*w*_ is the mass of water.

### Printing

A modular 3D printing test platform was used test different consistencies and printing parameters. The prototype is composed of two subsystems; the printer frame performing the Cartesian motion and a custom extruder. The test platform is described in further detail in the Supplementary Information.

Two different CAD-models were used in the printing trials. The first model was a 20 mm by 20 mm by 20 mm cube. The second model was a 14 mm long bone-like shape suitable for tensile testing, which is shown in the Supplementary Information Fig. S9. All measurement and analysis reported in this paper, apart from tensile measurements, is based on the cube geometry.

All printing trials were conducted in 25 °C and 50% relative humidity. The constant printing parameters are shown and described in Table 3. The calculations used to derive the parameters are shown in the Supplementary Information. The structures were printed on a fine wire mesh to enable moving them onto drying racks after printing.

**Table 3:** Process parameters tested.

Parameter name	Value	Unit	Description
Linear movement	8.00	mm/s	The linear movement speed of the extruder head
Extruder diameter	1.20	mm	The orifice diameter of the syringe tip (needle gauge: 16)
Layer height	1.00	mm	Distance incremented between each layer
Extrusion width	1.40	mm	The width of the non-circular printing path
Piston speed	0.05	mm/s	The speed of the syringe piston
Volumetric flow rate	0.56	ml/min	Volumetric flow rate, calculated from piston speed and syringe diameter
Layer infill	50.00	%	Amount of material used to fill the model volume
Perimeters	2		Number of external shells
Infill pattern	rectilinear		Pattern used in the infill
First layer height	1	mm	Distance between the syringe tip and printing platform during the first layer

EFCNF was printed at three different consistencies (15.5 wt%, 20 wt% and 25 wt%) and the first phase of the drying was performed in three different air drying conditions (T:25 °C,RH: 50%, T:50 °C, RH:50% and T:85 °C, RH:25%). 3 parallel prints from each printing parameter set were performed resulting in a total of 27 cubes. The nomenclature and parameters used for the specimens are shown in Table 4.

**Table 4:** Sample names based on consistency and drying conditions.

Name	Consistency (wt%)	Temperature (°C)	Relative humidity(%)
SC15.5T25RH50	15.5	25	50
SC15.5T50RH50	15.5	50	50
SC15.5T85RH25	15.5	85	25
SC20T25RH50	20	25	50
SC20T50RH50	20	50	50
SC20T85RH25	20	85	25
SC25T25RH50	25	25	50
SC25T50RH50	25	50	50
SC25T85RH25	25	85	25

The printed structures were dried in a controlled temperature and humidity chamber (Memmert GmbH, Germany). Samples were kept in the chamber for a minimum of 5 h. After this initial drying, samples were left to dry at 25 °C and 50% RH for a minimum of 30 h. Samples were weighed approximately once an hour with a precision scale (Mettler, OH, USA) during the initial drying. Once removed from the chamber the samples were weighed approximately once every 10 h until the mass reached a steady state. The printed structures do not reach a fully dry state due to, e.g., residual and ambient moisture. We establish an approximate drying time with an exponential decay model fit performed on the drying data. In the context of research, we established this limit as the theoretical dry weight of the object with an addition of a 5% margin. The drying curves as well as related calculations are described in further detail in the Supplementary Section of this paper.

### Scanning and point cloud analysis

The 3D printed cubes were scanned with a structured-light 3D scanner with a rotating scanning platform (Einscan SP, China). Each 3D-print was scanned in three different orientations and 12 different rotational positions of the scanning platform. The resulting 36 individual point clouds were combined into a single point cloud using the EinScan host software. The point clouds were exported both in an ASCII point cloud (.asc) and a stereolithography (.stl) format from which the dimensional data was acquired.

Point clouds were analyzed using CloudCompare. Firstly, the six planes constituting the box-like shape were determined with the random sample consensus (RANSAC) point cloud shape detection algorithm^44^. Secondly, the distance between these planes was used to create a geometrical primitive box with the same dimensions. Thirdly, the box was registered to the point cloud by initial manual alignment followed by a fine registration with an iterative closest point (ICP)-algorithm. Finally, the mesh to cloud distance was computed between the created box and the point cloud. The process is illustrated (Fig. S5) and described in further detail in the Supplementary Section.

### X-ray microtomography (XµCT)

The imaging is based on X-ray attenuation maps (shadowgrams) that are obtained from multiple positions around the sample. By using an inverse Radon transform the original 3D structure of the sample can be calculated from the series of the shadowgrams^45^. As the X-rays are sensitive to the density of the material, also the resulting 3D image matrix represents the density differences in the sample. The typical imaging volume for common XµCT devices is from few cubic millimeters to tens cubic of centimeters. The best spatial resolution for table-top XµCT being currently below 1 µm. The obtained 3D digitalized fiber networks can be computationally analyzed by various ways^46^. The possibilities include formation, density, shape, orientation, thickness, pore size distribution, pore/fiber connectivity and so on.

To determine the porosity of an XµCT data we utilize the fact that the XµCT system measures the X-ray abrsorption coefficients of the sample in 3D, including the interior of the sample. In the final 3D image, the X-ray absorption coefficients are remapped into gray values in the 3D matrix, each cell (referred to as a voxel) having also a known volume. In case of binary structures, containing only air (void) and some other material (solid), we can separate the void and solid by choosing a threshold value for the X-ray absorption coefficient, i.e., gray value thresholding the 3D image. We also need to define the area in 3D space containing only the sample. This procedure is called segmentation, and in a binary case the output of this procedure is called a mask. The porosity can be defined by dividing the number of void voxels by the total number of voxels inside the mask. The limitation of the procedure is the spatial separation abilities of the XµCT system. The main factors affecting the spatial separation are the sample size/pixel size, geometry of the XµCT system and imaging noise.

A total of three different cubes were imaged using XµCT; one cube from sample sets SC15.5T50RH50, SC20T50RH50 and SC25T50RH50.

### Tensile testing

Tensile testing pieces were printed with all three consistencies; 15.5 wt%, 20 wt% and 25 w%. All tensile testing specimens were air dried in a temperature of 50 °C and a relative humidity of 50%. The samples were sanded flat prior to testing to enable more secure attachment to the tensile testing device. Each tensile testing piece was measured with a digital caliper (Mitutoyo, Japan) and weighed with a precision scale (Mettler, OH, USA) prior to tensile testing. The densities of the tensile testing pieces were calculated based on these measurements. The tensile testing geometry is shown in the Supplementary Section Fig. S11.

The specimens were kept at 25 °C and 50% relative humidity for a minimum of 24 h prior to testing and weighed prior to testing. An Instron 4204 Universal Testing System equipped with a 1 kN load cell operating in tensile mode was utilized to analyze the tensile properties of the 3D prints. Measurements were performed in quintuplicate at an extension rate of 1 mm min^−1^.

## Supplementary information

Supplementary information

## Data Availability

The datasets generated during and/or analyzed during the current study are available from the corresponding author on reasonable request.
