# Author Correction: Shape fidelity and structure of 3D printed high consistency nanocellulose

**DOI:** 10.1038/s41598-020-58231-z

**Published:** 2020-01-24

**Authors:** Ville Klar, Jaakko Pere, Tuomas Turpeinen, Pyry Kärki, Hannes Orelma, Petri Kuosmanen

**Affiliations:** 1 Aalto University School of Engineering, Department of Mechanical Engineering, Aalto, 00076 Finland; 2 VTT Technical Research Centre of Finland Ltd, Espoo, 02044 Finland

Correction to: *Scientific Reports* 10.1038/s41598-019-40469-x, published online 07 March 2019

In the Supplementary Information file originally published with this Article, the equations for the plots were erroneous. These errors have been corrected in the Supplementary Information that now accompanies the Article.

Additionally, this Article contained a typographical error in the Methods section under subheading ‘Printing.’ where,

“We establish an approximate drying time with a one term polynomial fit performed on the drying data.”

now reads:

“We establish an approximate drying time with an exponential decay model fit performed on the drying data.”

This error has now been corrected in the HTML and PDF versions of the Article.

