# Supplementary material for: Shape fidelity and structure of 3D printed high consistency nanocellulose
Source: Sci Rep. 2019 Mar 7;9:3822. doi: 10.1038/s41598-019-40469-x (PMC6405753; doi:10.1038/s41598-019-40469-x)
Supplement: Supplementary file 1 — Supplementary information [file 41598_2019_40469_MOESM1_ESM.pdf]

Shape fidelity and structure of 3D printed high consistency nanocellulose

Ville Klar[1,*], Jaakko Pere[2], Tuomas Turpeinen[2], Pyry Kärki[1], Hannes Orelma[2], and Petri Kuosmanen[1]

[1]Aalto University School of Engineering, Department of Mechanical Engineering, Aalto, 00076, Finland
[2]VTT Technical Research Centre of Finland Ltd, Espoo, 02044, Finland
[*]ville.klar@aalto.fi

SUPPLEMENTARY INFORMATION

Keywords: Additive manufacturing, nanocellulose, shape fidelity, enzyme

3D printer testing platform

Samples were printed with a pupose-built modular DIW test platform. The platform consists of two mechatronic subsystems; a custom extruder (Fig S1a) and the printer frame performing the Cartesian motion (Fig S1b).

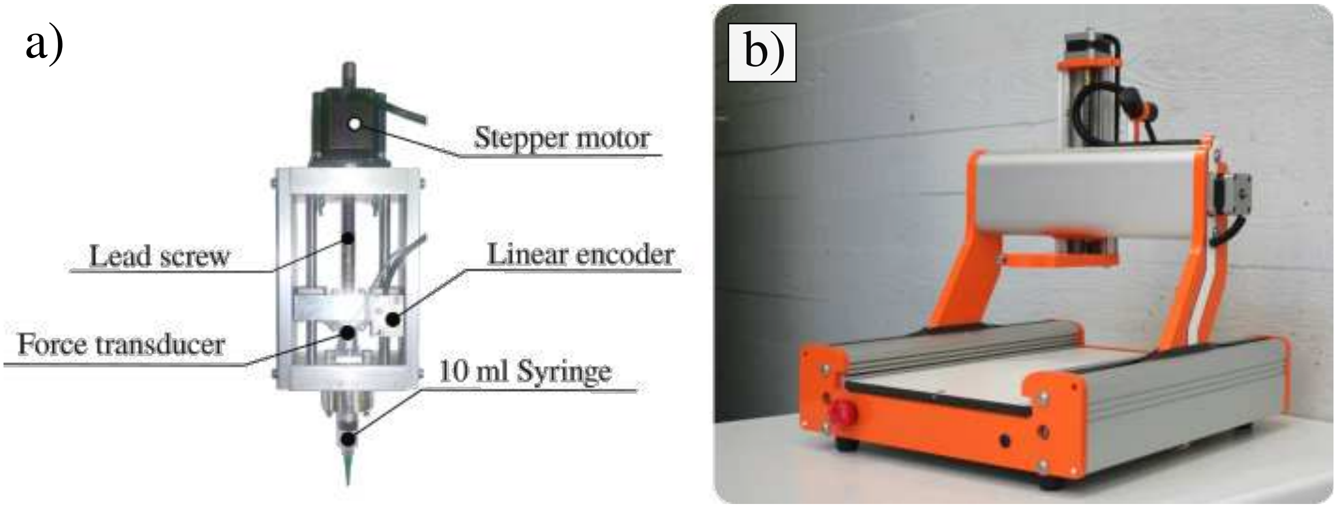

Figure S1. a) Extruder b) Printer frame

The frame is a table-top CNC-system (Stepcraft, Germany). [1] The printer's stock control electronics were replaced with an Arduino Mega development [2] connected to a RAMPS1.4 [3] shield. The Marlin firmware was used to move the stepper motors based on gcode input from the serial bus. [4] The gcode was produced with Slic3r [5] and sent to the printer via Pronterface.[6] The extruder is attached to the printer with the standard Ø 43 mm tool holder.

The extrusion is based on a syringe mechanism, i.e., flow is produced by actuating a syringe piston. The extruder design is based on standard 10 ml luer lock syringes (Henke Sass Wolf, Germany). The plastic syringe piston was replaced with an aluminium version to avoid errors from buckling and elastic deformation.

[1]Stepcraft 210,https://stepcraft.odoo.com/shop/product/20442-stepcraft-1-d-210-desktop-system-kit-190?category=26
[2]Arduino Mega, https://store.arduino.cc/arduino-mega-2560-rev3
[3]RAMPS 1.4 https://reprap.org/wiki/RAMPS$_1$.4
[4]Marlin, http://marlinfw.org/, https://github.com/MarlinFirmware/Marlin
[5]Slic3r, http://slic3r.org/
[6]http://www.pronterface.com/

The syringe piston is actuated with a linear stepper motor (OMC Stepperonline, China). A miniature load cell (S2TECH, U.S.A) was installed between the linear stepper motor screw and syringe piston to measure the piston force. The load cell readings are influenced by the friction between the syringe gasket and wall. An approximate gasket friction force was measured under zero-load conditions and subtracted from the net force. The linear movement of the syringe piston mount was measured with magnetic linear encoder (Renishaw, U.K.). The extruder was controlled and measured with a National Instruments data acquisition device (DAQ) and Labview. The linear movement was controlled with closed loop PID control scheme. The extruder signals from the RAMPS board were acquired with the DAQ and used to start and stop extrusion according to the gcode.

Printing parameters

In this study we used a 1.2 mm diameter tip, 1.4 mm layer width, a 1 mm layer height and an 8 mm/s printing speed. The 1.2 mm tip was chosen as it was the smallest standard tip size through which all three different solids content were extrudable.

The Slic3r manual recommends extrusion widths ranging from 1.05 to 1.7 times the tip diameter for FFF printing. [7] In this study we used an extrusion width of 1.4 which is 1.167 the used tip diameter. In FFF printing, layer heights typically range between 0.25 to 0.5 times the tip diameter. Such small values do not work in DIW as the solidification is slow and excessively small layer heights result in overfilling. A sufficiently large layer height is necessary to prevent the extruder head from colliding with the previously printed sections. A 1 mm layer height was chosen based on preliminary testing.

The cross-sectional area of the print path is calculated in Slic3r from layer height and extrusion width The slicing algorithm approximates the profile as a rectangle with semi-circular ends. The area of such a shape is

$$A_{path} = (w-h)h + \pi(\frac{h}{2})^2 \quad (1)$$

, where A_{path} is the cross-sectional area, w is the extrusion width, and h is the layer height. By knowing the area of the cross-section and the scanning speed of the printhead v_{print}, the optimal volume flow for the chosen parameters is

$$Q = A_{path} * v_{print} \quad (2)$$

Based on equations 1 and 2 the target volumetric flow rate, with the chosen layer width and height is $0.56899\frac{ml}{min} \approx 0.57\frac{ml}{min}$.

If the extrudable medium is assumed incompressible, the volume flow can be calculated from the speed of the plunger with equation

$$Q = \frac{v_{piston}\pi d^2}{4} \quad (3)$$

, where v_{piston} is the piston's linear speed and d is the inner diameter of the syringe. The nominal inner diameter of the Henke Sass Wolf 10 ml luer lock syringes is 16 mm. Thusly we can solve for a target piston speed,

$$v_{piston} = \frac{4Q}{\pi d^2} \quad (4)$$

which yields $0.047165\frac{mm}{s} \approx 0.05\frac{mm}{s}$

[7] http://manual.slic3r.org/advanced/flow-math

Drying times

The drying times are calculated from the mass measurements performed during the drying. A non-linear least squares analysis is used to fit a first order polynomial to the measurement data. The drying time is interpolated with the resulting function.

This threshold is set at the point where the mass of the construct corresponds with a structure with less than 5 % residual moisture. This point was calculated using using equation

$$m_{95} = \frac{C_m \cdot m_w}{0.95} \tag{5}$$

, where C_m is consistency of the material (0.155, 0.2 or 0.25) and m_w is the wet mass of the printed construct. This calculation was performed on each sample individually. These consistency values were established with the dry mass measurements described in the Methods - section of the main article.

Each sample set is plotted in the same subplot and each figure consists of three subplots that show the fitting for the different initial solids contents. Figures S2, S3 and S4 show the results for samples dried at T:25 °C & RH: 50% , T:50 °C & RH: 50% and T:85 °C & RH: 25% respectively.

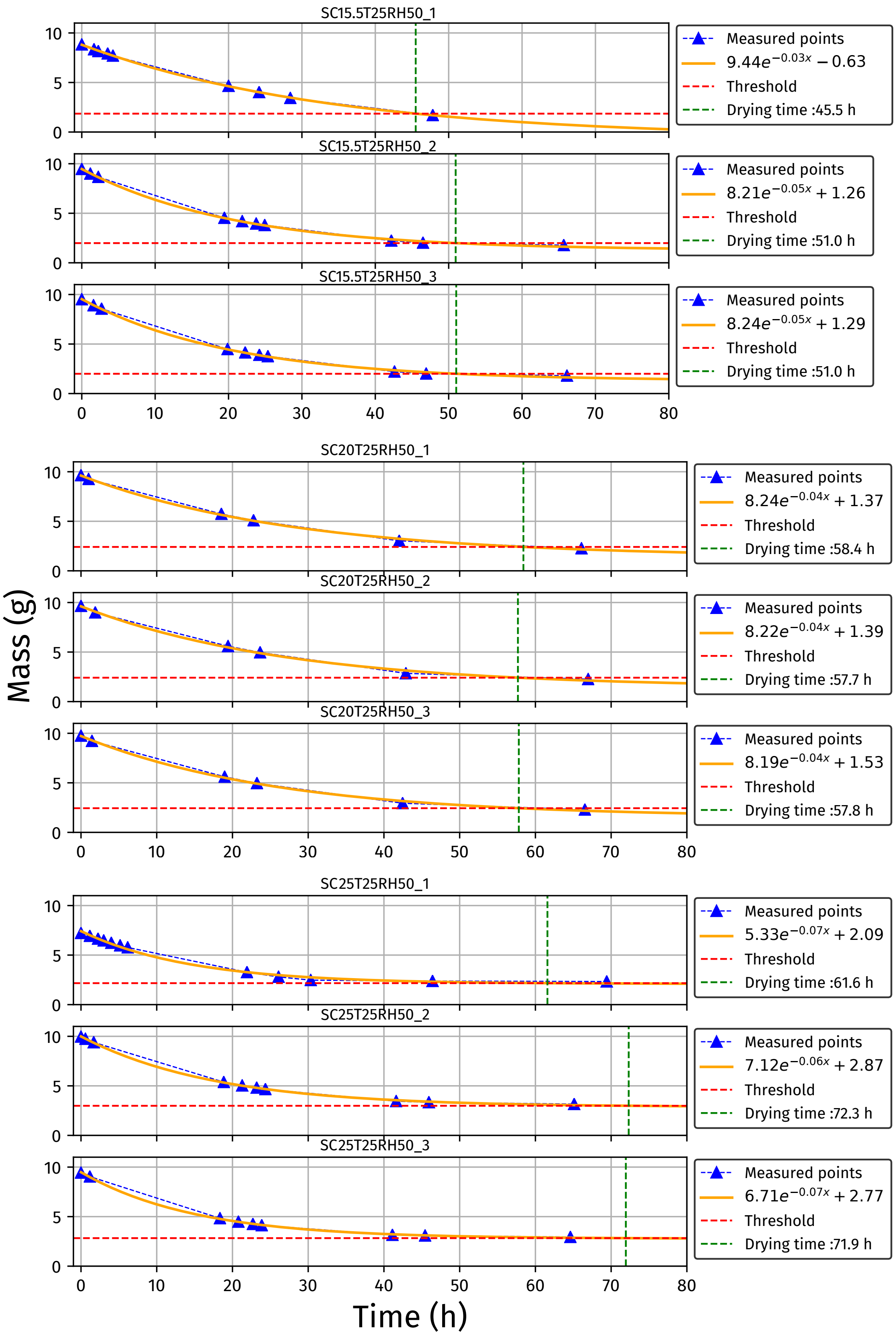

Figure S2. T: 25 °C, RH: 50%

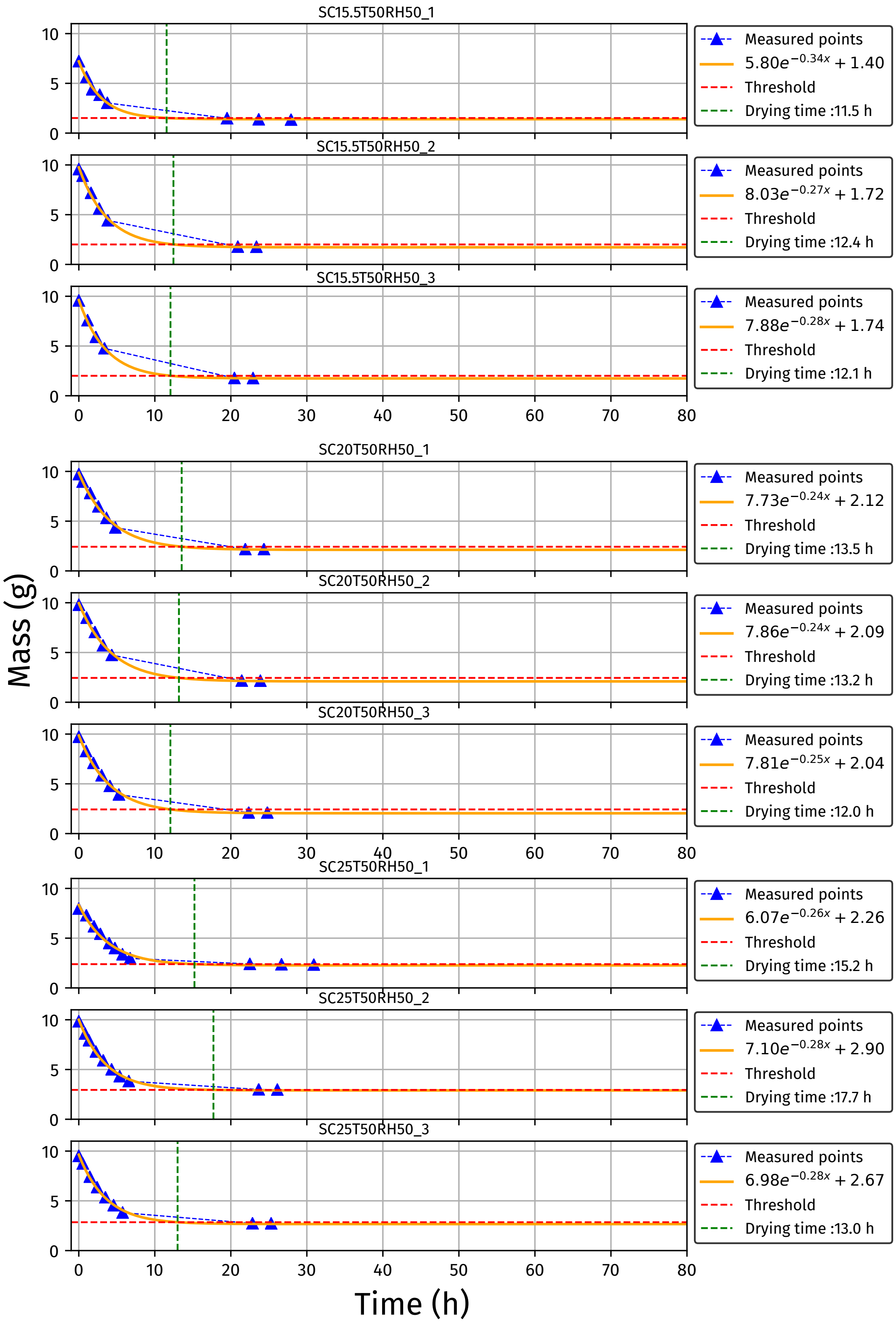

Figure S3. T: 50 °C, RH: 50%

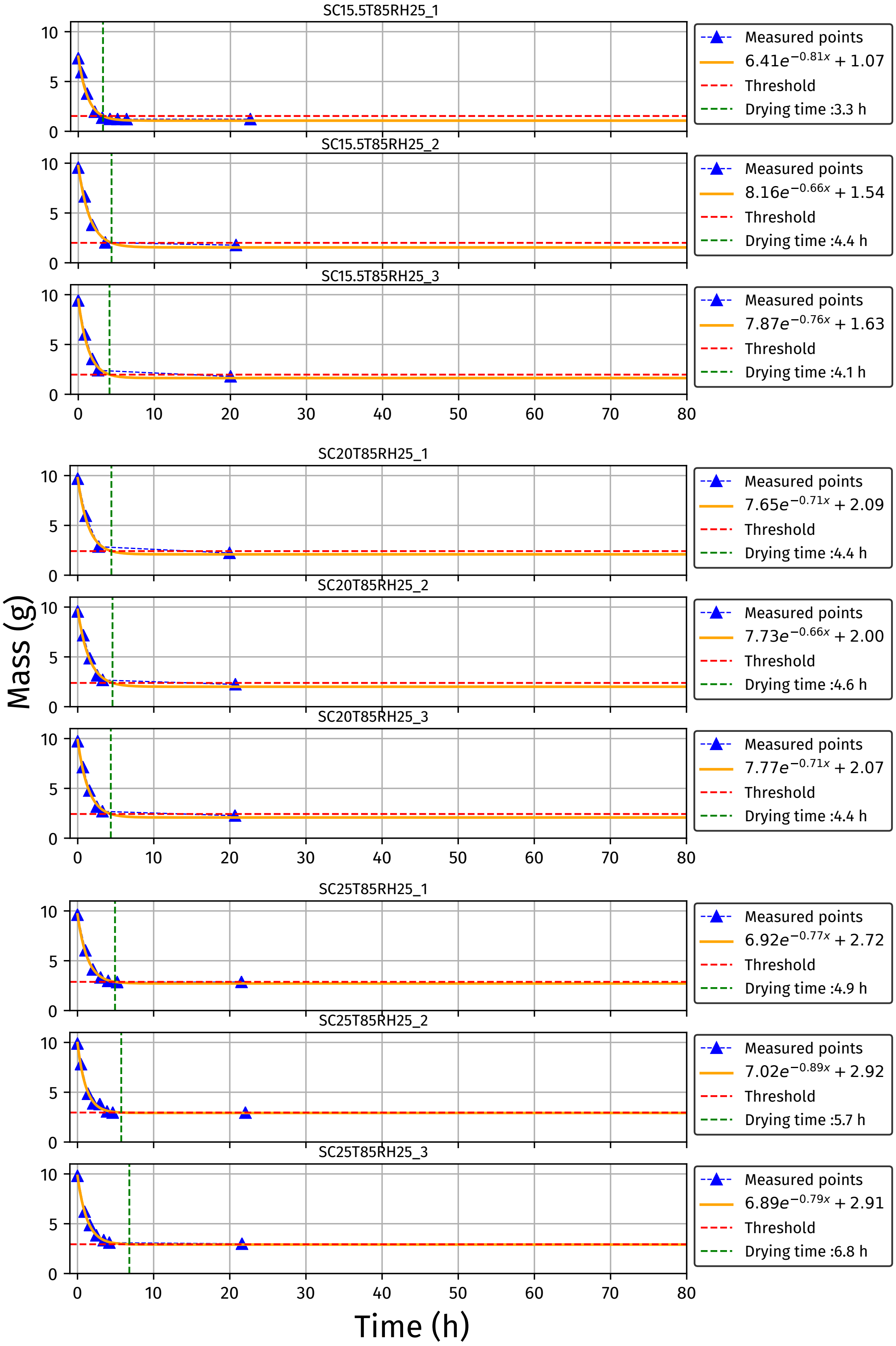

Figure S4. T: 85 °C, RH: 25%

Point cloud data

In this research, each sample was scanned a total of 36 times (3 orientations, 12 angles of rotating table) and the resulting scans were combined by the EinScan software into a single point cloud. The point cloud size is dependent on the print size with approximately 220 000 , 330 000 and 440 000 points for cubes printed from 15.5, 20 and 25 –w% suspensions respectively. The point clouds have a mean surface point density of approximately 500 points per mm^2. However, the surface point density is not constant and appears to have a bimodal distribution with a standard deviation of approximately 300 points per mm^2.

The acquired point clouds were analyzed using CloudCompare. [8] The point cloud analysis consists of three phases: plane fitting, registering and mesh to cloud comparison. Firstly, the RANSAC shape detection algorithm is used on the point cloud (Fig S5a) to fit planes on each face of the scanned object (shown if Fig.S5b). 5000 was used as the minimum amount of points per plane. The fitted planes are then used to measure the distance between opposing faces to establish the dimensions of the rectangular construct in X, Y and Z directions. The mean dimensions between the detected planes are used to construct a geometrical primitive. Secondly, the geometrical primitive and the point cloud are registered. They are first registered manually followed by fine registration using CloudCompare's iterative closest point (ICP) algorithm. A point cloud with the registered primitive is shown in Fig. S5c. Finally, the distance between the original point cloud and registered primitive is computed and visualized with a false color image as shown in Fig. S5d. Volumes for the scanned objects were calculated using the numpy-stl Python libary. [9]

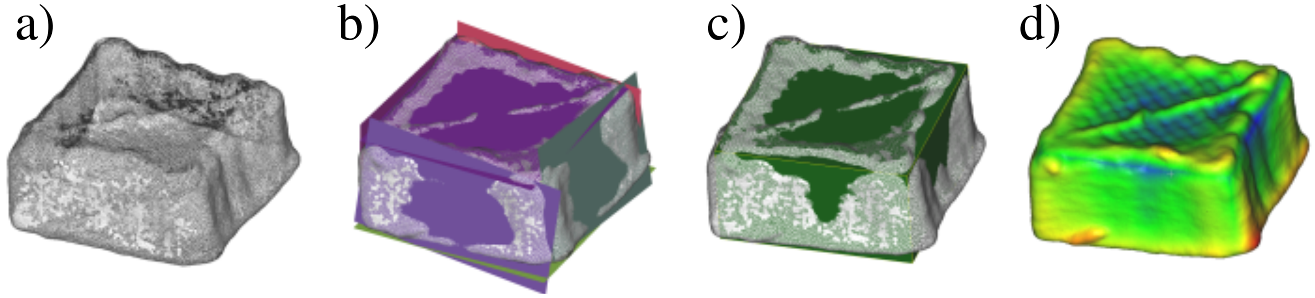

Figure S5. Point cloud analysis process.
a) Point cloud b) Detected planes c) Fitted shape primitive d) Error between fitted primitive and point cloud

The manufacturer (EinScan) reports a resolution of 0.17 mm - 0.2 mm and a single-shot accuracy (±0.05mm) for the utilised 3D scanner. [EinScan SP](https://www.einscan.com/einscan-se-sp) These values refer to the resolution and accuracy obtained by a singular scan of an object. As the point clouds used for analysis in this research are not from singular point cloud, these values cannot be directly used incorporate instrument error into the measurements.

To establish an estimate of error regarding scanning and shape analysis, we performed an uncertainty quantification with reference geometry (a wooden die). This reference geometry was chosen because it has similar optical properties and dimensions as the printed constructs. Furthermore, similarly to the printed constructs it is not a perfect cube because of the chamfered corners. This enables estimation of the error regarding the shape fitting. The die and scanned geometry are shown in Figure S6a and Figure S6b respectively.

[8] CloudCompare website, https://www.cloudcompare.org/
[9] numpy-stl project page,https://pypi.org/project/numpy-stl/

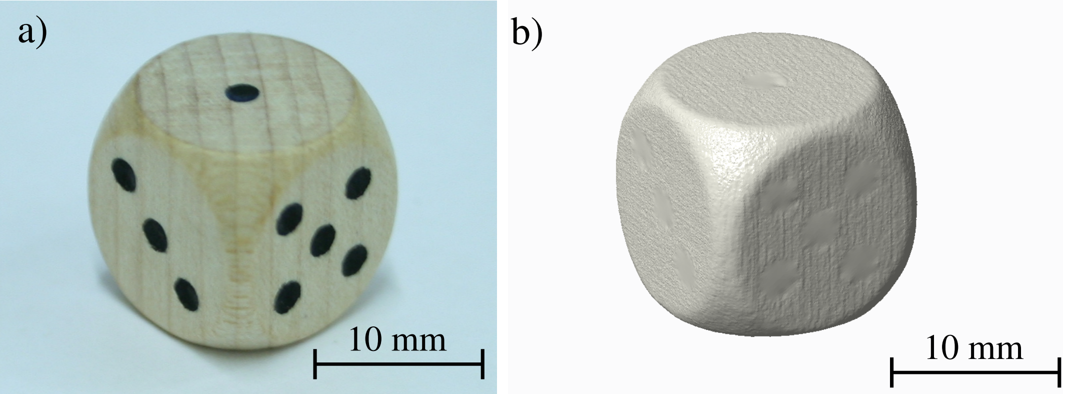

Figure S6. a) Wooden die used as reference geometry, b) Scan of reference geometry

The die was scanned 10 times and each resulting point cloud was analysed using the shape quantification method demonstrated in this research. We also measured the die with a 599-100 Micromaster Micrometer (Brown & Sharpe, Switzerland). The mean value, standard deviation and relative error from micrometer measurements is shown in Table S1.

Table S1. Dice measurements

	V (mm^3)	X (mm)	Y (mm)	Z (mm)	RMS (mm)	d (mm)	$SD_d(mm)$
Mean	2581.698	14.121	13.947	13.889	0.263	0.088	0.248
Standard deviation	6.480	0.017	0.020	0.024	0.006	0.004	0.005
Micrometer measurement	-	14.141	13.941	13.865	-	-	-
Relative error (%)	-	-0.140	0.042	0.176	-	-	-

The results shown in Table S1 indicate that errors from the shape quantification and scanning are negligible in comparison to the standard error calculated from the parallel measurements (Table 1) performed on different samples.

All 27 printed cubes were 3D scanned. Samples printed with solids contents 15.5 wt%, 20 wt% and 25 wt% are shown in figures S7, S8 and S9 respectively. Each cube is represented as a color image where red color denotes positive error (outside of the fitted shape) and blue denotes negative error (inside the fitted shape). The distance between the point cloud and the fitted shape is also plotted in the bar graph below each color image. The x-axis shows the distance and the y-axis shows the number of points within this distance measurement. A narrower distribution means that more points are within a small distance of the box-like fitted shape. Higher volume prints have a higher number of points in the measured point clouds. As a consequence, the bar graphs do not share the same y-axis scaling.

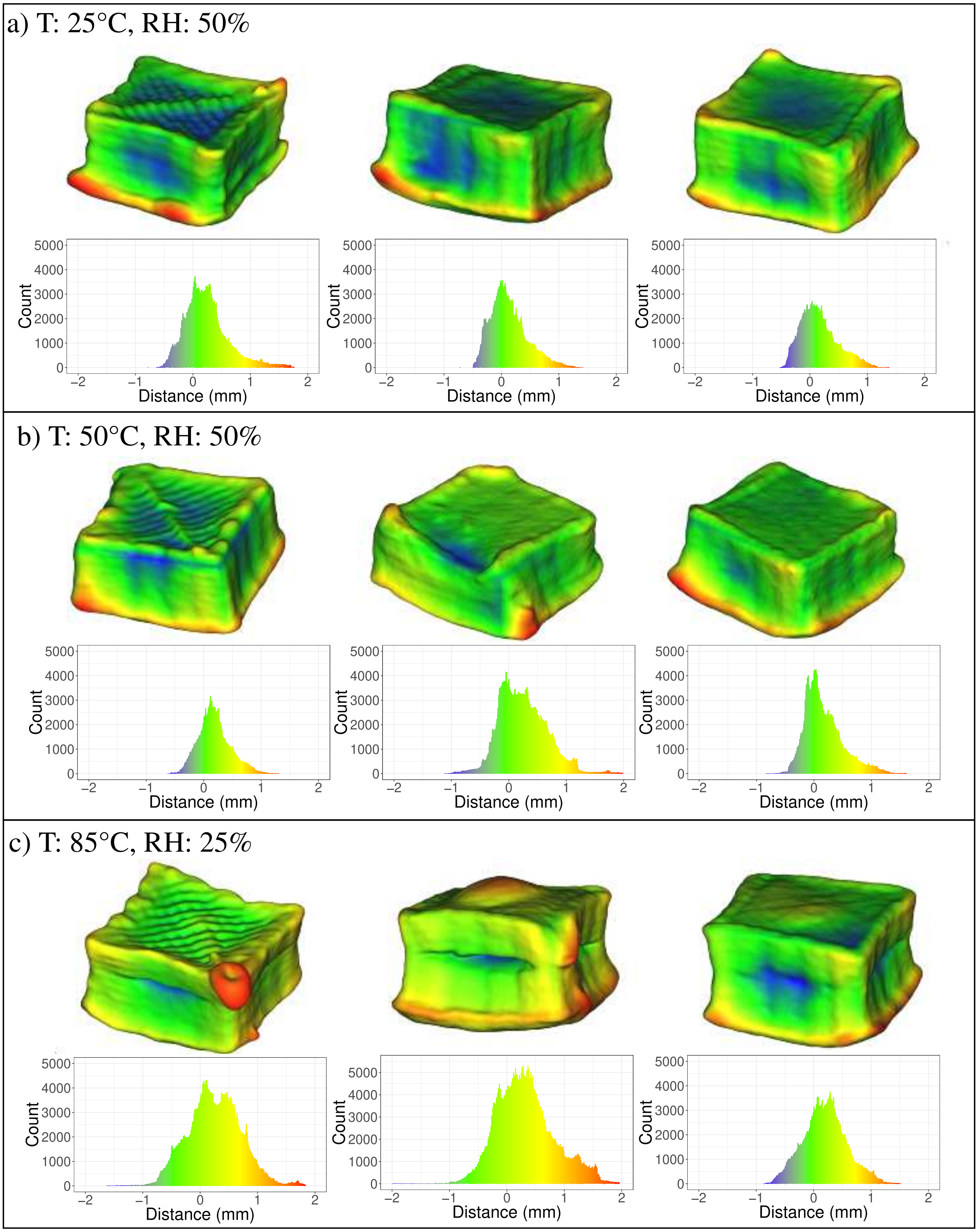

Figure S7. SC:15.5 wt%

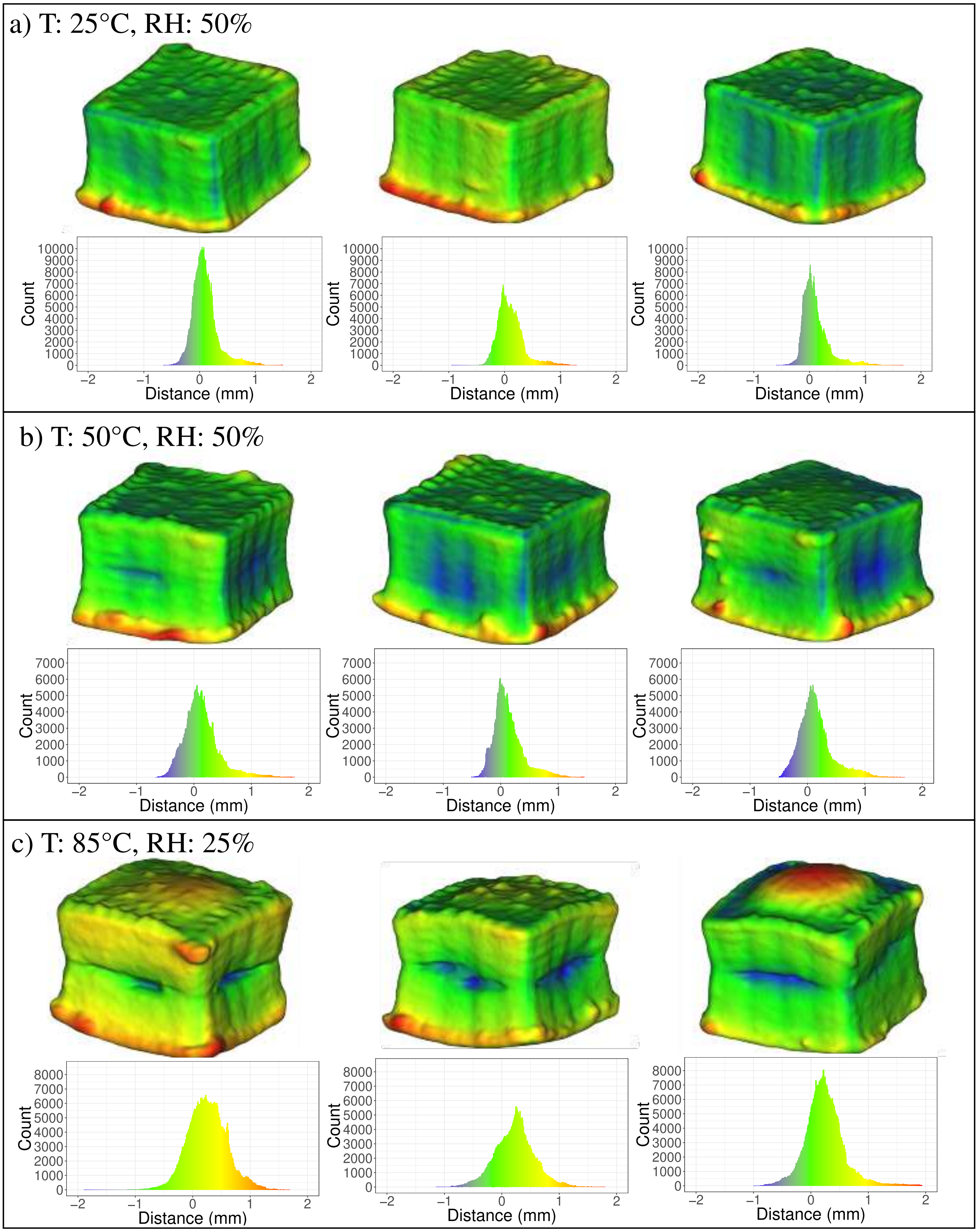

Figure S8. SC:20 wt%

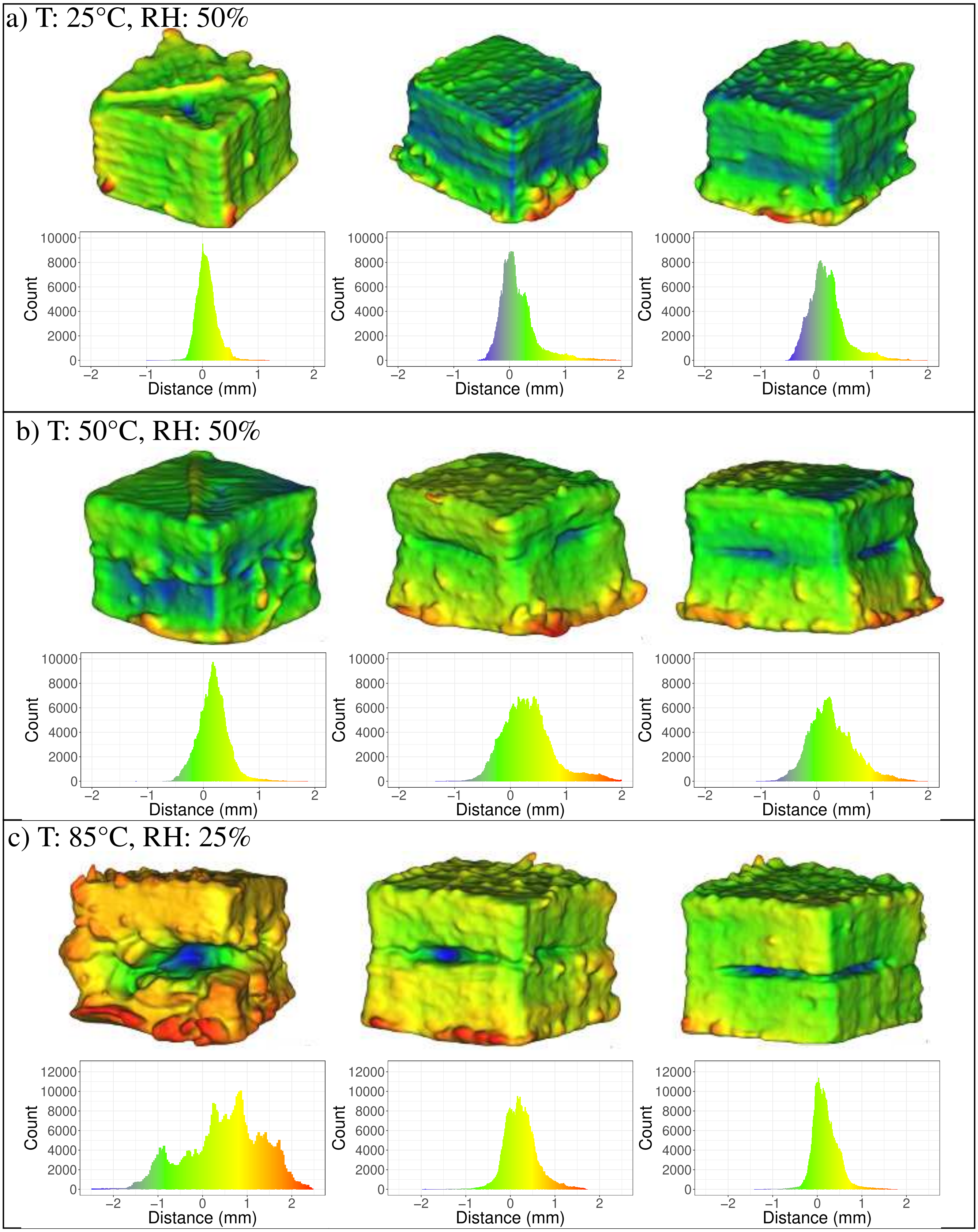

Figure S9. SC:25 wt%

Tensile testing

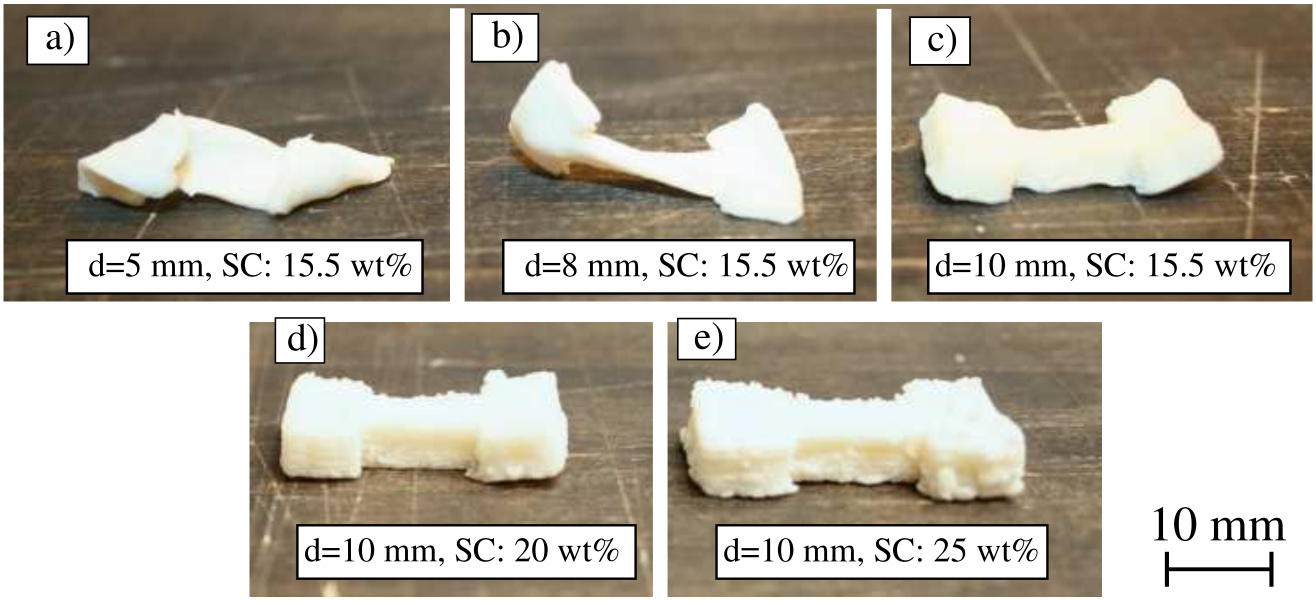

Figure S10. Influence of solids content (SC) and tensile test piece model thickness (d) on warping
a) d:5 mm SC:15.5 wt%, b) d:8 mm SC:15.5 wt%, c) d:10 mm SC:15.5 wt%, d) d:10 mm SC:20 wt%, f) d:10 mm SC:25 wt%

Figure S10 shows the influence of solids content and model (CAD-data) on warping of the geometry. The warping was not quantitatively measured with tensile testing geometries but observations indicate that warping attenuates with both model thickness and solids content. Based on the preliminary testing a tensile geometry with a thickness of 12 mm and length of 22 mm was set. The geometry is shown in figure S11.

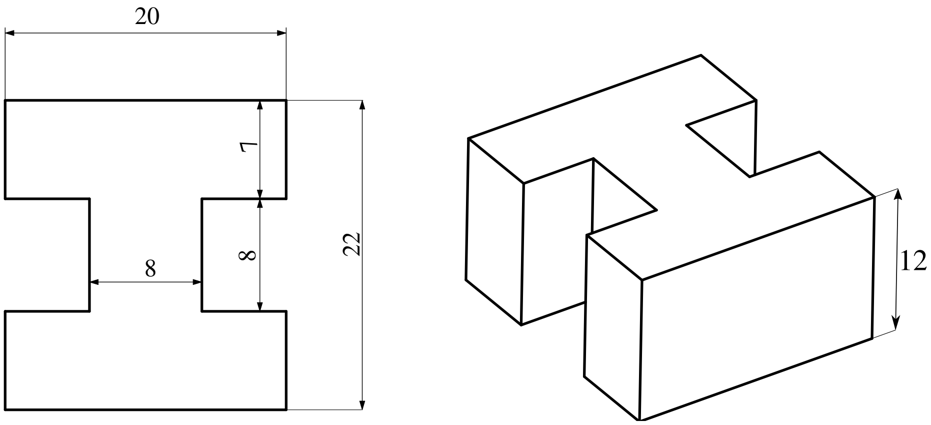

Figure S11. Tensile testing geometry

While increasing the thickness of the prints attenuated the warping, the top and bottom surfaces of the tensile testing pieces were not flat. Consequently, they were sanded flat prior to testing. Unsanded samples would fracture when attached to the tensile testing machine. After sanding each specimen was measured separately and the stress and elongation values were calculated based on individual specimen dimensions (area and length). The dimensions of each tensile testing specimen (after drying) are shown in table S2 Future research should address warping and aim for the use of standard tensile testing geometries. The authors also consider it likely that cracks and other imperfections may be introduced to the specimens during sanding or attachment into the tensile testing machine. Such imperfections are likely to reduce the measured load at break.

Table S2. Dimensions of tensile testing specimens

	Area (mm^2)	Length under tensile testing (mm)	Total mass of specimen (g)
SC15.5 sample: 1	15.5	3.1	0.6
SC15.5 sample: 2	15.8	3.1	0.7
SC15.5 sample: 3	17.0	3.4	0.7
SC15.5 sample: 4	17.5	3.5	0.8
SC15.5 sample: 5	17.1	3.4	0.8
SC20 sample: 1	26.6	5.0	1.1
SC20 sample: 2	28.6	5.0	1.1
SC20 sample: 3	26.5	5.0	1.1
SC20 sample: 4	21.4	3.9	0.8
SC20 sample: 5	27.4	5.0	1.1
SC25 sample: 1	37.1	6.0	1.4
SC25 sample: 2	36.6	5.8	1.3
SC25 sample: 3	36.0	5.9	1.4
SC25 sample: 4	34.6	5.5	1.3
SC25 sample: 5	39.8	6.2	1.5

The stress elongation curves for the tested samples are shown in figure S12.

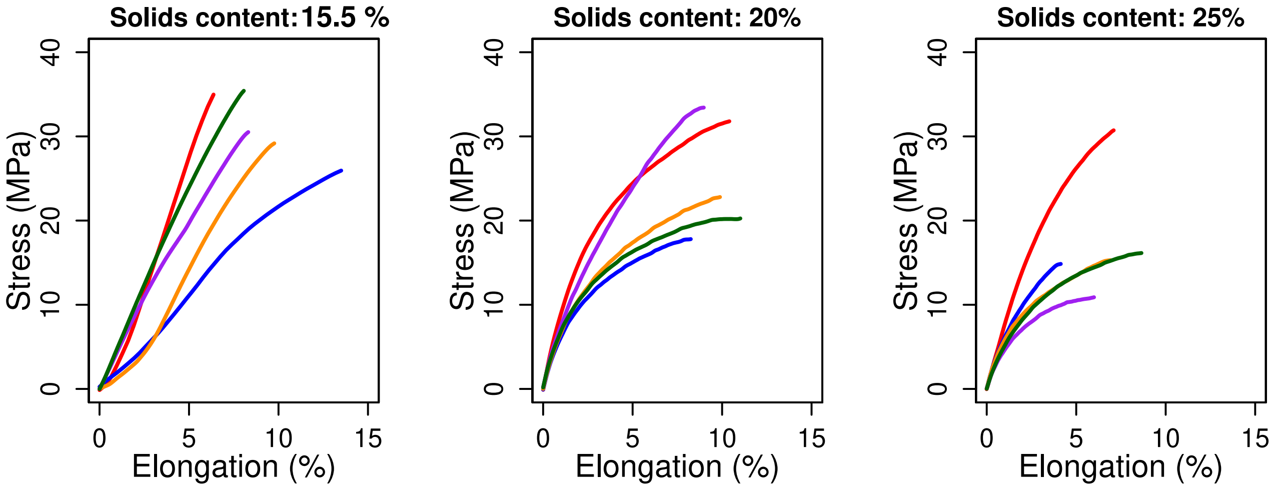

Figure S12. Stress strain
